# A flowgraph model for bladder carcinoma

**DOI:** 10.1186/1742-4682-11-S1-S3

**Published:** 2014-05-07

**Authors:** Gregorio Rubio, Belén García-Mora, Cristina Santamaría, José Luis Pontones

**Affiliations:** 1 Instituto de Matemática Multidisciplinar, Universitat Politècnica de València, España; 2 Departamento de Urología, Hospital Politécnico La Fe, Valencia, España

**Keywords:** Flowgraph model, Bladder carcinoma, Erlang distribution

## Abstract

**Background:**

Superficial bladder cancer has been the subject of numerous studies for many years, but the evolution of the disease still remains not well understood. After the tumor has been surgically removed, it may reappear at a similar level of malignancy or progress to a higher level. The process may be reasonably modeled by means of a Markov process. However, in order to more completely model the evolution of the disease, this approach is insufficient. The semi-Markov framework allows a more realistic approach, but calculations become frequently intractable. In this context, flowgraph models provide an efficient approach to successfully manage the evolution of superficial bladder carcinoma. Our aim is to test this methodology in this particular case.

**Results:**

We have built a successful model for a simple but representative case.

**Conclusion:**

The flowgraph approach is suitable for modeling of superficial bladder cancer.

## Background

Bladder tumors are a challenge in urology. They pose an important public health problem because they are biologically very aggressive and are highly prevalent in western countries. Approximately 75-85 % of patients with newly diagnosed bladder carcinoma have non muscle-invasive bladder carcinoma (NMI-BC), which can be managed with transurethral resection (TUR). TUR is a surgical endoscope technique used to remove the macroscopic tumor from the interior of the bladder. However it has a notable tendency to recur (30-85 %) and less frequently to progress to muscle invasive stages (10-20 %). The object of this study is the NMI-BC, that makes up 70 % of the total health care cost of this disease. A review about the NMI-BC may be found in [1].

Biotechnological advances have allowed us to use different therapeutic procedures (surgery, radiotherapy, chemotherapy, immunotherapy) successfully but still many patients suffer an unfavourable outcome without control of disease. In practice urologists have a serious problem: some patients with similar characteristics undergo different evolution. Consequently, this creates a problem as to the choice of treatment to be applied. Urologists need tools to accurately predict the real evolution of the disease, that help them to improve treatment modalities and follow-up schemes of non-muscle invasive bladder cancer patients. In this regard an important contribution [2] appeared in European Urology, the official journal of the European Association of Urology. By means of looking up tables the probability of recurrence and progression for a patient is provided. However only time to first recurrence is considered, and the analysis is reduced to the Cox proportional hazards regression model. Later works have studied the model validation, finding some limitations [3].

Our team has been working with urologists from University Hospital La Fe for the last ten years. We have developed several models trying to capture different aspects of the disease evolution. Our aim for the near future is to detect the most relevant predictive factors, and also to perform an accurate model of the disease evolution. The first objective includes investigating at the genetic and molecular level, while the second one could be achieved with a suitable multistate model. While the process may be reasonably modeled by means of a Markov process, in order to more completely model the evolution of the disease this approach is insufficient. Specifically, it is possible that time spent in a state influences the future evolution of the process, i.e., it not only depends on the current state. The semi-Markov framework allows a more realistic approach, but calculations become frequently intractable. In this context, flowgraph models provide an efficient approach for the analysis of time-to-event data, since their introduction in this field a few years ago [4]. The present work is a first step in order to explore the evolution of the recurrence progression process by means of this methodology.

The paper is organized as follows: first we review a few basic concepts of survival analysis, phase-type distributions and Erlang distributions, needed to build the model. Then we present the essentials of flowgraph models and important features of our approach. The section that follows deals with a simple flowgraph model for the recurrence-progression process in NMI-BC, constructed using a database from La Fe University Hospital of Valencia (Spain). Finally, some conclusions are discussed.

## Survival analysis and phase-type distributions

### Survival analysis

Survival analysis techniques deal with the analysis of data taking times from a well-defined *time-origin *until the occurrence of some particular event or *end-point *.

To summarize survival data there are two key functions: the Survival Function and the Hazard Function. Let T be the random variable associated with the survival time (time until the ocurrence of the event).

The Survival Function is

S(t)=P(T≥t)=1-F(t)

where *F*(*t*) is the distribution function of T. It expresses the probability that an individual survives from the time origin to some time beyond *t*.

The Hazard Function is given by

$$\lambda \left(t\right)=\underset{\text{Δ}t\to 0}{\text{lim}}\frac{\text{P}\left(t\leq \text{T}<t+\text{Δ}t|\text{T}\geq t\right)}{\text{Δ}t},$$

which expresses the hazard rate or the instantaneous event rate.

In survival analysis data are frequently censored [5], which means that the event of interest has not been observed. The follow-up time of those patients must be taken into account, because it informs us of the fact that the individual has been free of event until the present moment. For instance we started with 957 patients, of whom 434 underwent a recurrence, 24 a progression, and 499 had censored times, which means that at the time of their last check-up they had no recurrence or progression.

### Phase-type distributions

In order to model lifetimes, mixtures of distribution functions are useful. In this context phase-type distributions [6] are very interesting, because of their properties and they provide computations with manageable analytical expressions. Let us summarize the main concepts: the distribution *F*(-) on [0, ∞) is a phase-type distribution (PH-distribution) with representation (*α, T*) if it is the distribution of the time until absorption in a Markov process on the states {1, . . . , *m, m *+ 1} with generator

$$\left(\begin{array}{c} T\;T^{0}\\ 0\;\;0 \end{array}\right),$$

and initial probability vector (*α, α*_*m*+1_) where *α *is a row m-vector.

The matrix *T *of order *m *is non-singular with negative diagonal entries and non-negative off-diagonal entries, *T*^0 ^is a column matrix with nonnegative entries, and it holds that

$$-Te=T^{0},$$

where *e *denotes a column vector with all components equal to one.

The distribution *F*(-) is given by

(1) F(t)=1-α exp(Tt)e,t≥0

and the density *f*(*t*) by

$$f\left(t\right)=\alpha \text{exp}\left(Tt\right)T^{0}.$$

The survival function is

(2) S(t)=α exp(Tt)e

and the hazard function is given by

$$h\left(t\right)=\frac{\alpha \text{exp}\left(Tt\right)T^{0}}{\alpha \text{ exp}\left(Tt\right)e}.$$

Finally, the Laplace transform is

(3) $$L\left(s\right)=\alpha_{m+1}+\alpha {\left(sI-T\right)}^{-1}T^{0},\;\text{for}\;Re\left(s\right)>0.$$

Phase-type distributions are a closed class for finite mixtures, and form a class weakly dense in the class of general distributions defined on the positive real line.

A particular case of phase-type distribution, relevant in our approach, is the Erlang distribution. An Erlang distribution *E*[*r, λ*] has a representation (*α, T*) as a phase-type [7]:

$$\alpha ={\left(1,0,\ldots ,0\right)}_{1\times r}$$

$$T={\left(\begin{array}{ccccc} -\lambda & \lambda & & & \\ & -\lambda & \lambda & & \\ & & \ddots & \ddots & \\ & & & -\lambda & \lambda \\ & & & & -\lambda \end{array}\right)}_{r\times r}$$

A finite mixture of Erlangs distributions is therefore a phase-type distribution. We are interested in the class of mixtures of three Erlang distributions studied in [8]. The distribution function of the elements in this class is given by the expression

(4) $$G\left(t\right)=p_{1}F_{1}\left(t\right)+p_{2}F_{2}\left(t\right)+p_{3}F_{3}\left(t\right),$$

with *p*_1 _+ *p*_2 _+ *p*_3 _= 1, *p_i _*> 0, *i *= 1, 2, 3.

Let us denote the three Erlangs by *E*[*r*_1_, *µ*_1_], *E*[*r*_1_, *µ*_1_], *E*[*r*_1_, *µ*_1_], with *µ_i _*> 0 and *r_i _*a positive integer, *i *= 1, 2, 3. In the particular case with *r*_1 _= 1, *r*_2 _= 3, *r*_3 _= 5 the representation as phase-type distribution is (*α, T*) where

(5) $$\alpha =\left(p_{1}\;p_{2}\;0\;0\;p_{3}\;0\;0\;0\;0\right)$$

(6) $$T=\left(\begin{array}{ccccccccc} -\mu_{1} & 0 & 0 & 0 & 0 & 0 & 0 & 0 & 0\\ 0 & -\mu_{2} & \mu_{2} & 0 & 0 & 0 & 0 & 0 & 0\\ 0 & 0 & -\mu_{2} & \mu_{2} & 0 & 0 & 0 & 0 & 0\\ 0 & 0 & 0 & -\mu_{2} & 0 & 0 & 0 & 0 & 0\\ 0 & 0 & 0 & 0 & -\mu_{3} & \mu_{3} & 0 & 0 & 0\\ 0 & 0 & 0 & 0 & 0 & -\mu_{3} & \mu_{3} & 0 & 0\\ 0 & 0 & 0 & 0 & 0 & 0 & -\mu_{3} & \mu_{3} & 0\\ 0 & 0 & 0 & 0 & 0 & 0 & 0 & -\mu_{3} & \mu_{3}\\ 0 & 0 & 0 & 0 & 0 & 0 & 0 & 0 & -\mu_{3} \end{array}\right)$$

The versatility of distributions given in (4) let have us several options to fit them to our interest distributions. The way we tried was to perform some experimental computations, considering different values of *r*_1_, *r*_2 _and *r*_3_. Mixture (5) was explicitly given in [8], and we found it worked very well.

## Flowgraph models

A flowgraph model is a graphical representation of a multistate model that consists of directed line segments (*branches*) connecting the states, namely, a directed graph. The branches are labeled with *transmittances*, that are the transition probability *p_ij _*from state *i *to state *j *multiplied by an integral transform *G_ij_*(*s*) of the transition time probability density function (PDF). This transformation can be a characteristic function (CF), a moment generating function (MGF), a Laplace transform (LT), or even an empirical transform [9][10]. Flowgraphs are used to represent semi-Markov processes, given that allowed waiting time distributions go beyond the exponential distribution directly linked to Markov processes.

**Figure 1:**
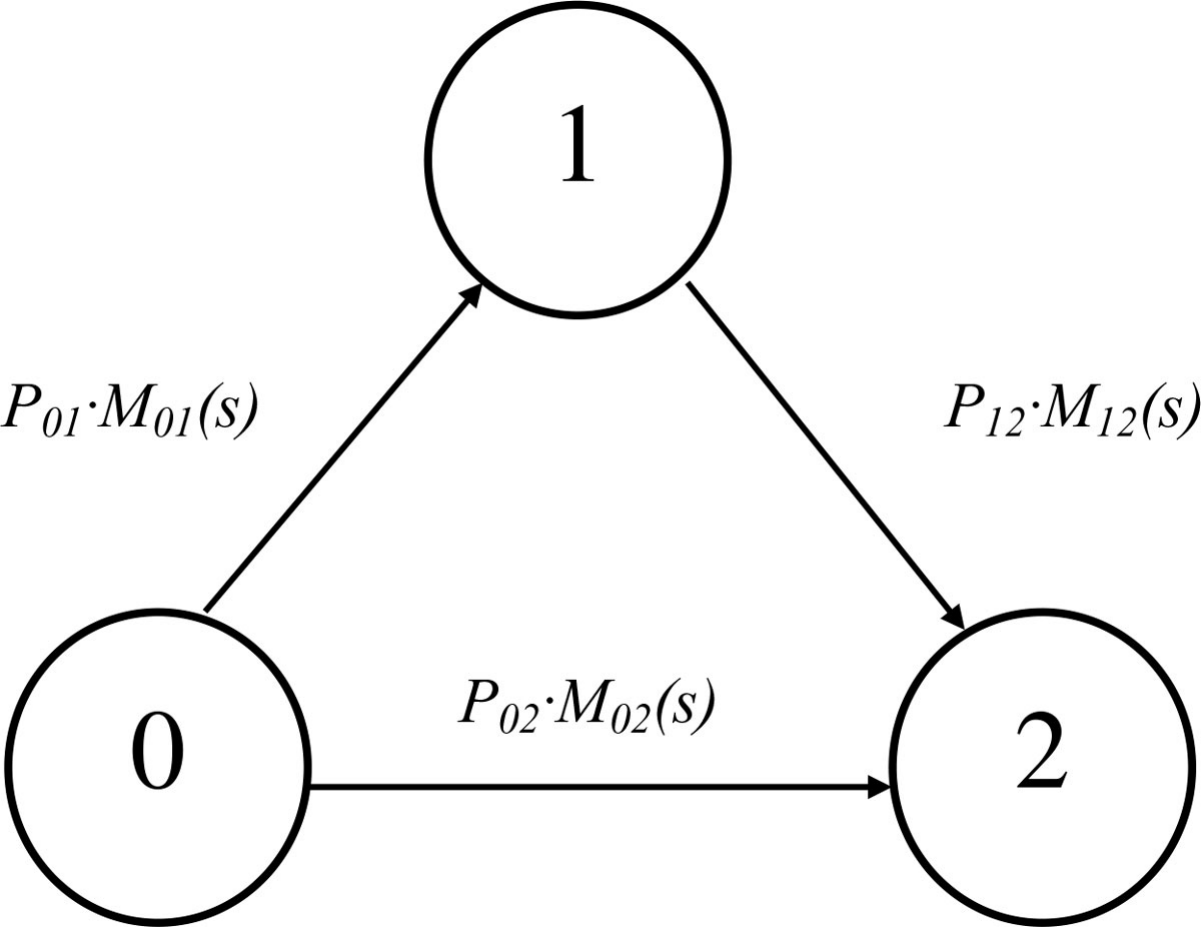
**Three-state illness-death model**.

For instance, Figure 1 shows the flowgraph of the three-state illness-death model that we will use in this paper, based on [11].

Transmittances are combined according to a systematic procedure (see [12], section 2.5), in order to compute the transforms for the transitions of interest. For instance, the rules pertaining to the graph in the Figure 1 are the following:

1) The transmittance of transitions in series is the product of the series transmittances.

2) The transmittance of transitions in parallel is the sum of the parallel transmittances.

These rules are applied later in building the model.

In order to perform the model, the first step is to select a suitable distribution for the waiting time in each transition. Our approach will be to compute the empirical distributions (Kaplan-Meier [5]) and approximate them using mixtures of Erlang distributions. Specifically we use the mixture given by (5)-(6). Note that the cumulative distribution function is easily computed from expression (1). The parameters *p_i _*and *µ_i _*are calculated by minimizing

(7) $$||F_{ij}\left(t\right)-G_{ij}\left(t\right)||,$$

where *F_ij _*is the empirical distribution for the transition ij and *G_ij _*the mixture distribution for the same transition. Initial values for the minimization process are needed. In order to estimate these values (and also to decide a suitable mixture, in our case (5)-(6)) we use a non-negative least squares fit (Lawson-Hanson algorithm [13]).

More precisely, the idea is the following. Based on [8], we try several Erlang distributions in expression (4). Given *F*_1_, *F*_2 _and *F*_3_, and an empirical distribution *F *we consider the system

$$\begin{array}{c} F=p_{1}F_{1}+p_{2}F_{2}+p_{3}F_{3}\\ 1=p_{1}+p_{2}+p_{3} \end{array}$$

which we fit by non-negative least squares, to compute *p*_1_, *p*_2 _and *p*_3_. In this way we obtain reasonable initial values for the parameters *p_i _*and *µ_i_*.

Once the parametric distributions have been computed, the Laplace transforms are easily calculated from (3). Then we compute the Laplace transform relevant to the transitions of interest, applying the above rules. The final step is to invert these transforms to obtain PDFs, for which we use an inversion algorithm called EULER, developed by Abate and Whitt [14].

Flowgraph models for stochastic networks were introduced by Butler and Huzurbazar [4]. An account of the theory developed up to 2005 may be found in [12]. A recent contribution proposing a prognostic model is [15].

## A flowgraph model for bladder carcinoma

### Data

The database was obtained from La Fe University Hospital of Valencia (Spain). It records clinical-pathological information from 957 patients, followed between January 1995 and January 2010. The primary tumor is a NMI-BC, which means that it is categorized as stage Ta or T1, according to the World Health Organization (WHO) TNM classification staging system [16]. After removal of the tumor by TUR, it may recur at a similar stage, which we call recurrence; or it may progress to muscle invasive stages T2, T3 or T4, which we call progression. The data record several recurrence times. This means that some patients have no recurrence at all, some have one or more recurrences, and some have progression (directly of after some recurrence). In our model we have considered progression and one recurrence. As stated above, 434 patients underwent a recurrence, 24 a progression, and 499 had censored times. Then, 63 patients were lost. From the remaining 371 patients, 17 underwent a progression, 226 a recurrence and times of the remaining 128 patients were censored. A full description of data may be found in [17].

### Flowgraph model

Our aim in this paper is to test the flowgraph methodology in this particular problem, and so we perform the simple model of Figure 1. In state 0 the patient is free of disease, after the TUR of the primary tumor. State 1 is the first recurrence, and state 2 is progression. Time is given in years.

By way of example, we are going to model the overall risk of progression. So we are interested in finding the probability distribution of time to reach state 2 for the first time starting in state 0, irrespective of the path that was taken. That is to say, the first passage distribution of going from disease free to muscle invasive stages. But the aim of a more general flowgraph model for the recurrence - progression process would be to predict the risk of recurrence or progression from any state.

**Figure 2:**
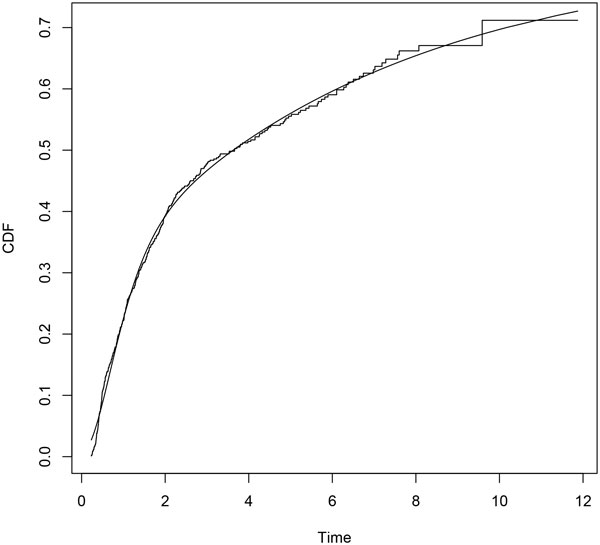
**Erlang mixture (*smooth line*) and empirical distribution (*step function*) for transition 01**.

**Figure 3:**
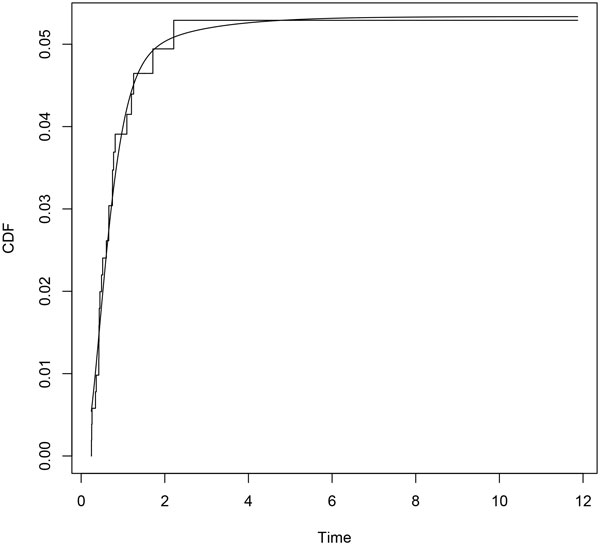
**Erlang mixture (*smooth line*) and empirical distribution (*step function*) for transition 02**.

**Figure 4:**
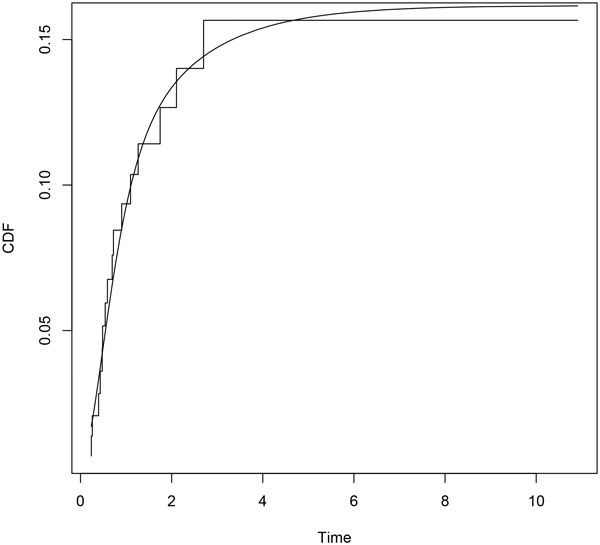
**Erlang mixture (*smooth line*) and empirical distribution (*step function*) for transition 12**.

Parametric distributions for all transitions and their Laplace transforms are performed according to the procedure described above. Minimization is carried out by means of the *constrOptim *function, from the R Stats Package [18]. We use the euclidean norm in (7). Empirical and parametric distributions for each transition are shown in Figures 2, 3 and 4.

Let us calculate the first passage distribution of going from state 0 to state 2. For this we compute the Laplace transform of the time to progression. Applying the rules 1 and 2 above, it would be given by:

$$LT\left(s\right)=p_{01}p_{12}LT_{01}\left(s\right)LT_{12}\left(s\right)+p_{02}LT_{02}\left(s\right)$$

**Figure 5:**
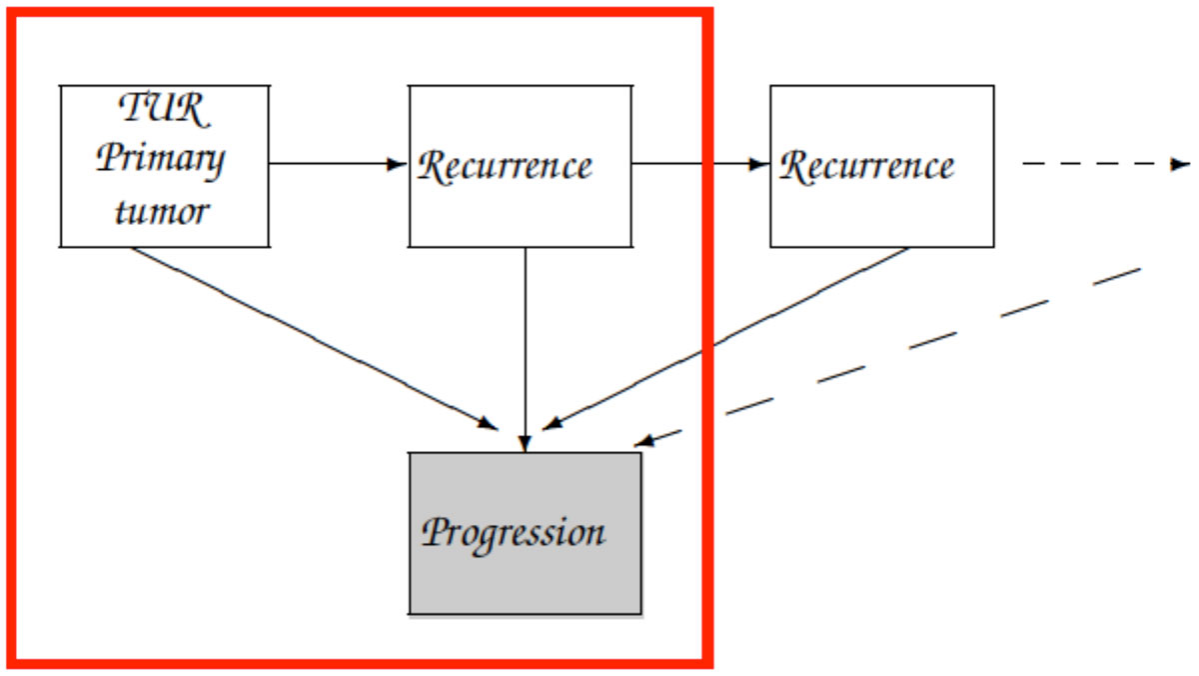
**Recurrence - progression process**.

However, it must be taken into account that our flowgraph is actually part of a more general graph that would model the disease process, see Figure 5. Passage from state 0 to state 2 is not certain to occur: a patient may only suffer recurrences, or even no recurrence. The probability of taking the considered path is *p*_01_*p*_12 _+ *p*_02_, and we must divide the preceding *LT*(*s*) by this probability to obtain the true Laplace transform [12, pag. 19]

$$LT\left(s\right)=\frac{p_{01}p_{12}LT_{01}\left(s\right)LT_{12}\left(s\right)+p_{02}LT_{02}\left(s\right)}{p_{01}p_{12}+p_{02}}$$

Probabilities *p_ij _*are assigned from estimations based on our data. They simply consist of the ratios between the number of progressions or recurrences and the number of patients who could undergo the relevant transition. Calculations are quite sensitive to these values. We tried with the current and also previous database. The best results were obtained taking *p*_01 _= 0.3967742, *p*_02 _= 0.02507837 and *p*_12 _= 0.03252033.

**Figure 6:**
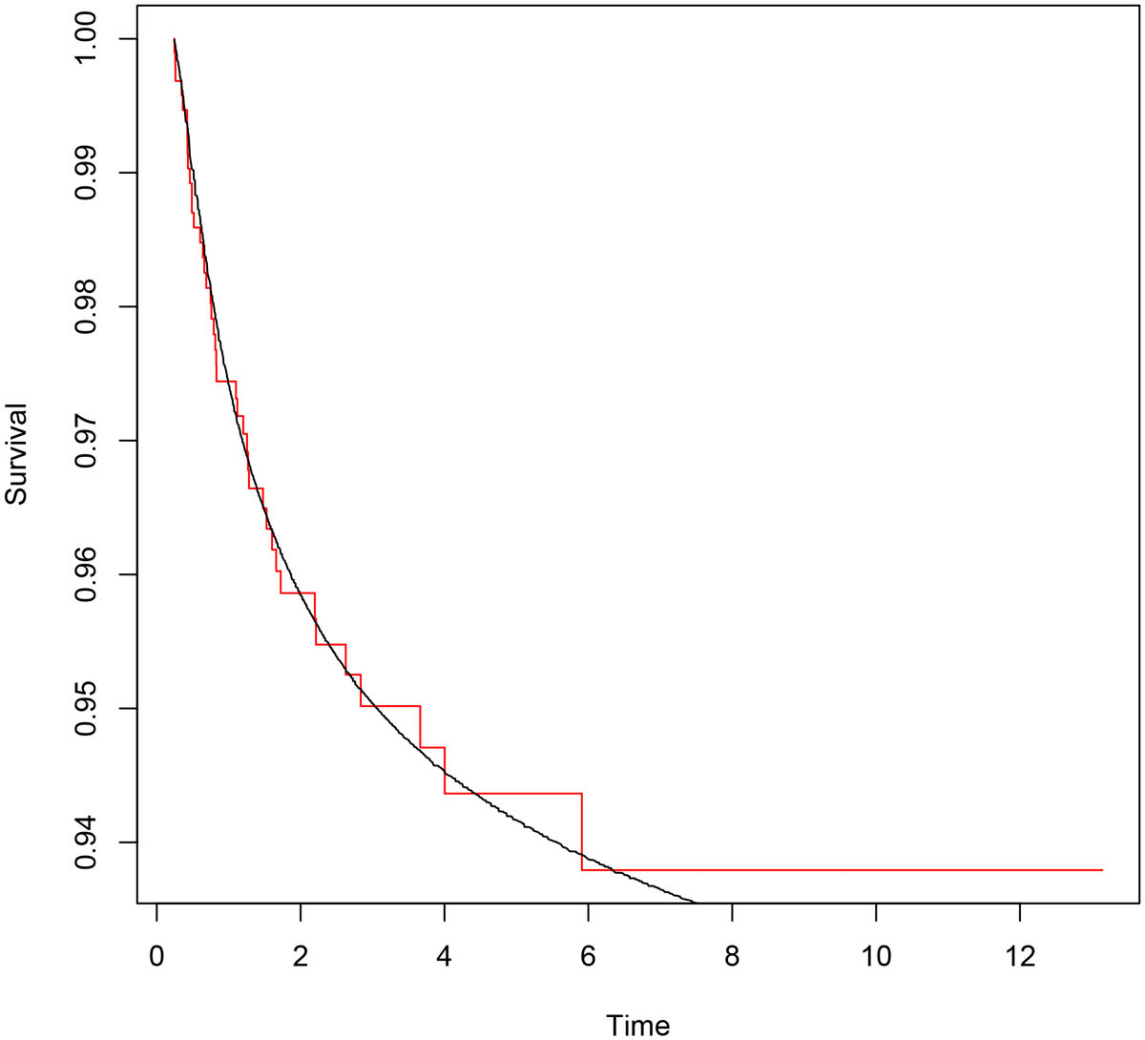
**Survival function model (*smooth line*) and empirical survival function (*step function*)**.

To recover the PDF we use a variant of the inversion algorithm EULER [15]. From this function we obtain the survival function (with regard to progression), that is shown in Figure 6, jointly with the empirical survival function. The hazard function may be also easily computed. Thus we have a parametric model to predict the probability of being free of progression at a given time. The procedure may be easily used to define risk groups, simply by calculating the survival functions of patients grouped according to common characteristics. Then the monitoring and treatment of patients can be adjusted according to their risk.

All computations were made in R. Besides the mentioned packages, we also used the expm [19], Matrix [20] and survival [21] packages.

## Discussion

A parametric approach in the framework of flowgraph models involves exploring parametric models looking for the distributions that match the data better. In [12] histograms of sample waiting times are suggested. In this paper we propose a fitting procedure using mixture of Erlang distributions. Figures 2, 3 and 4 show graphically that the fitted parametric distributions match the empirical distributions very well. Figure 6 shows that parametric distribution provided by the model matches also the empirical distribution of our interest transition very well.

**Figure 7:**
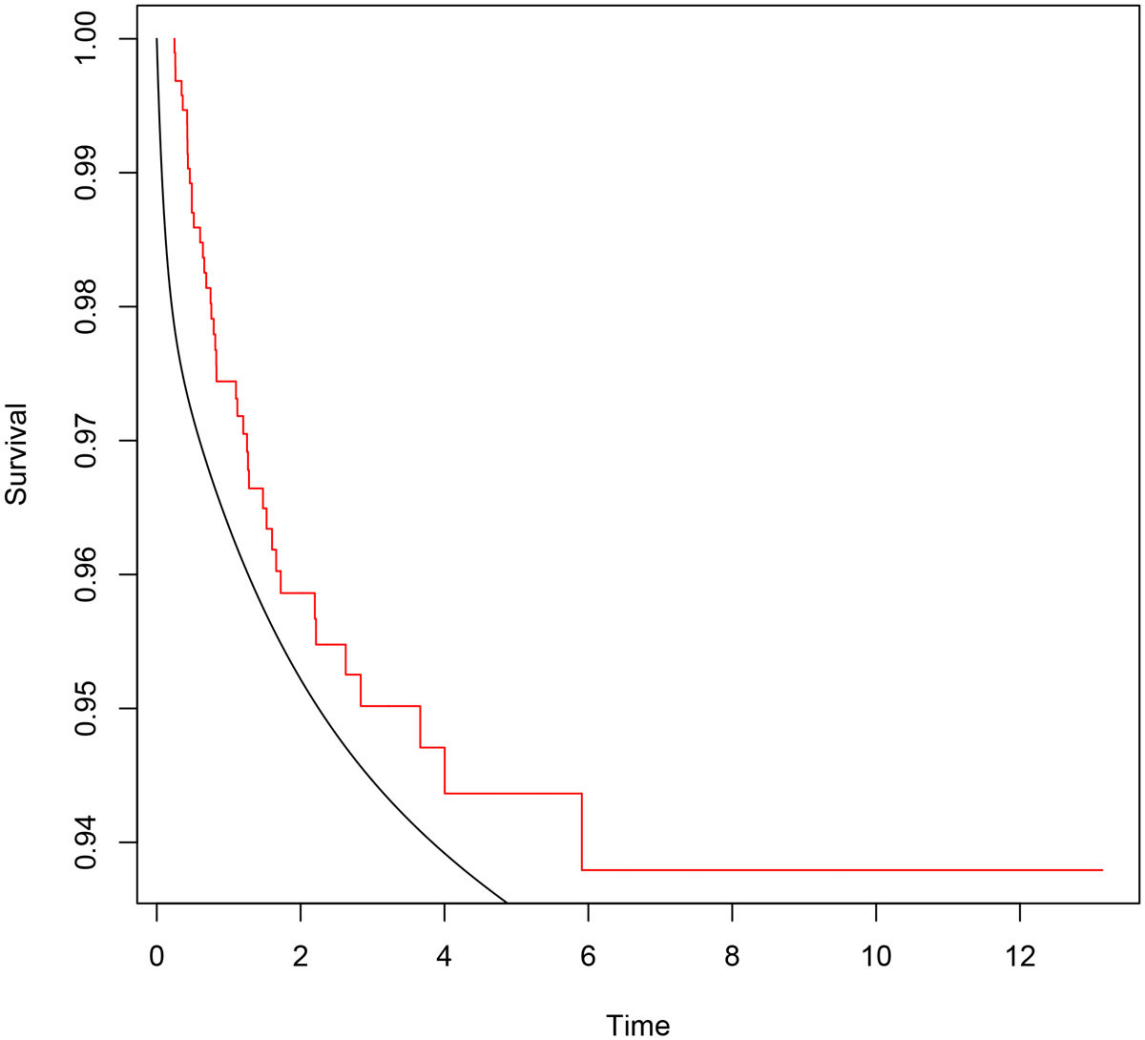
**Survival function of Markov model (*smooth line*) and empirical survival function (*step function*)**.

In order to contrast our results with other approaches, we have performed the classical multistate Markov model. The msm R package by C. Jackson [22] is a useful tool to manage Multi-state Markov and hidden Markov models in continuous time. Using this software, we found some similarities with our results, but overall they were worse, probably because the Markovian hypothesis is not satisfied. By way of example, Figure 7 corresponds to the first passage distribution of going from state 0 to state 2.

This is only a first step in applying the flowgraph approach to bladder carcinoma. Our aim is to incorporate covariates in a parametric model involving several recurrences and progression. Thus the doctors will have a useful tool to estimate the risk of recurrence and progression of patients according to their characteristics.

## Conclusions

These results suggest that the approach is suitable for modeling the evolution of the NMI-BC. Therefore it is justified to try to extend the model to more complex situations. Flowgraph methodology is very flexible. It allows the model to incorporate multiple recurrences, and recently also covariates [9]. Moreover non-parametric approaches are also available [10]. This versatility, along with the inclusion of molecular biomarkers, allow us to expect to get a very accurate model in a not too distant future.

## Competing interests

The authors declare that they have no competing interests.

## Authors' contributions

GR, BG and CS did the work of mathematical modeling. JLP addressed the medical context and obtaining tumor samples.

## Declarations

Publication of this article has been funded by Universitat Politècnica de València (Spain).

This article has been published as part of *Theoretical Biology and Medical Modelling *Volume 11 Supplement 1, 2014: Selected articles from the 1st International Work-Conference on Bioinformatics and Biomedical Engineering-IWBBIO 2013. The full contents of the supplement are available online at http://www.tbiomed.com/supplements/11/S1.
